# A novel method to eliminate the symmetry dependence of fiber coils for shupe mitigation

**DOI:** 10.1038/s41598-024-59330-x

**Published:** 2024-04-20

**Authors:** Tugba Andac Senol, Onder Akcaalan, Aylin Yertutanol, Ekmel Ozbay

**Affiliations:** 1 Department of Physics, Bilkent University, 06800 Ankara, Turkey; 2 Nanotechnology Research Center (NANOTAM), Bilkent University, 06800 Ankara, Turkey; 3 Department of Electrical and Electronics Engineering, Bilkent University, 06800 Ankara, Turkey; 4 Institute of Materials Science and Nanotechnology (UNAM), Bilkent University, 06800 Ankara, Turkey

**Keywords:** Optics and photonics, Physics

## Abstract

It is a well-known fact that interferometric fiber optic gyroscopes (IFOGs) are easily distorted by thermal effects and distortion results in the degradation of the performance of these sensors. Changing the fiber coil geometry, increasing the winding symmetry, adding fiber buffer layers around the fiber coil, using different modulation methods for multifunctional integrated optic chips, and using special types of fibers, such as photonic crystal fibers, are some alternative solutions for preventing this degradation. This paper, theoretically and experimentally, investigates not only how different types of fiber coil winding methods behave under different rates of temperature change but also presents a novel method, to the best of our knowledge, to eliminate the Shupe effect, without violating the simplest IFOG scheme. This method rules out the importance of the winding symmetry epochally and the need of any extra treatment for the fiber coil to increase the thermal performance of the system. Regardless of the symmetry of the fiber coil winding, the rate error due to the Shupe effect can be reduced to about $\pm 0.05^\circ /$h for any rate of temperature change with this new method according to the experimental results.

## Introduction

Interferometric fiber optic gyroscopes are used in inertial navigation systems both in industry and military fields requiring compact, cost-effective, and reliable solutions. They are used for guidance, navigation, and control in air and land vehicles. They use Sagnac Effect^1^. The improvements of low-loss optical fibers, as well as solid-state semiconductor light sources and detectors^2^ have helped the advent of the IFOGs in addition to the effect that Sagnac unearthed. Moreover, IFOGs are solid-state devices and have no moving parts, they have tendency for miniature manufacturing^3^, long lifetime^4^, very quick turn-on time^4^, high reliability^5–7^, high precision, and their sensitivity can be easily increased by increasing the number of wraps in the fiber coil used^5^. These are just some of the advantages that have ensured their outdistancing the other types of gyroscopes, e.g., ring laser gyroscopes (RLGs)^4,7–9^.

An ideal IFOG should be able to only measure the Sagnac phase shift. However, the Sagnac effect is not the only source of measurable phase shift in practice. Environmental effects such as temperature^10^, magnetic field^11,12^, or vibration^13^ create differences in the optical paths. These effects can cause some undesired nonreciprocal phases apart from the Sagnac phase shift. The real Sagnac phase shift can not be easily distinguished from the other nonreciprocal phase shifts. That’s why, they have to be minimised as much as possible in order to have a high quality fiber optic rotation sensor.

As the sensing element, the fiber coil takes the lead in affecting the performance of the IFOG. In particular, the thermal sensitivity of the fiber coil reveals its sensitivity. Special winding methods have been proposed to overcome this performance limitation^14–21^. The quadrupole winding pattern is one of the most widely used techniques with proven performance^14^. The counter-propagating light waves do not follow the same paths through the fiber segments in the fiber coil when they are exposed to different rates of temperature change. This causes a false rotation signal and the quadrupole winding pattern helps to reduce this non-reciprocal error. However, this winding method is no longer sufficient to meet the performance demands of IFOGs. Further work is required to increase the winding symmetry of the fiber coil and eventually improve the thermal performance of the IFOG^22^. Apart from the winding methods, mechanical studies^23^ and different modulation techniques^24,25^ have also been carried out to reduce the temperature effects. Using special types of fibers such as photonic crystal fibers (PCFs) in fiber coils to decrease the temperature dependency of the refractive index is also offered as an alternative to solve the problems caused by the environmental effects on IFOGs^26^.

In the present paper, we study the rate errors due to the thermal characteristics of different types of fiber coils both theoretically and experimentally. We bring in the trimming method in addition to the usage of different winding methods for the very same purpose. We verify the model experimentally as well with different rates of temperature change. Furthermore, we notice that the rate error due to the Shupe effect behaves independently in the direction of the real rotation during the temperature analyses. We develop a novel method in which the Shupe effect can be easily measured and integrated into the IFOG systems independent of temperature variation and fiber coil winding symmetry.

Beams traveling uneven paths create undesirable nonreciprocal phase shifts along with the real phase shift coming from the rotation that is to be measured. By considering this phase shift, the angular error of thermally induced non-reciprocity, $\phi _{shupe} (t)$, for the IFOG can be calculated by^10^

**Figure 1:**
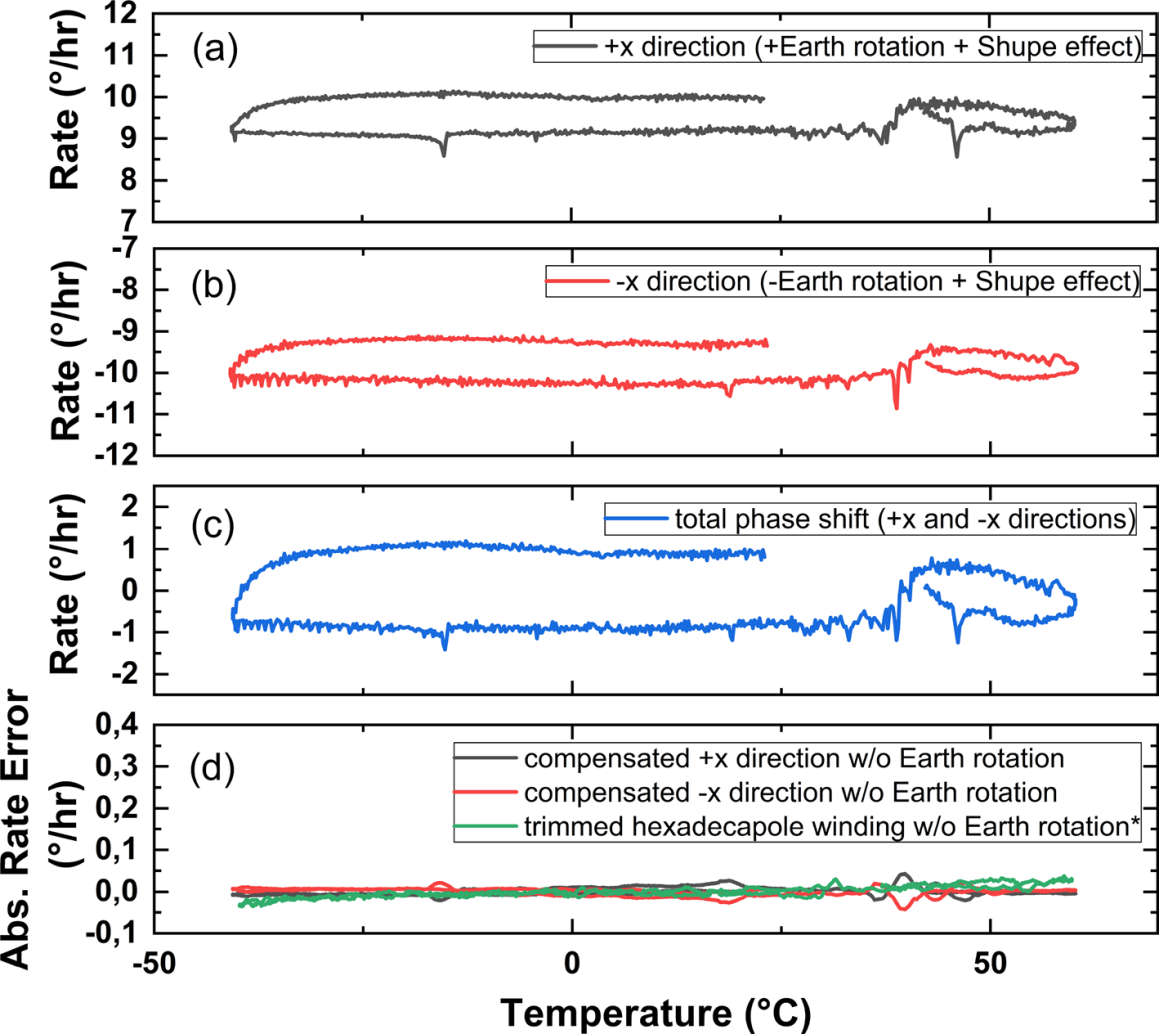
Rate versus temperature graph of the IFOG comprising the fiber coil with the hexadecapole pattern obtained at a rate of 0.2^∘^C/min temperature change horizontally placed in the (**a**) +x direction, (**b**) horizontally reversed placed in the $-$x direction, (**c**) total phase shift of the same IFOG in both directions, (**d**) compensated absolute rate errors in both directions compared with the result obtained from the IFOG comprising a perfectly trimmed hexadecapolar fiber coil at a rate of 0.2^∘^C/min temperature change.

1 $$\begin{aligned} \phi _{shupe} (t) = \frac{n_c}{4NA}(\frac{dn_c}{dT}+\alpha n_c)(\int _{0}^{L/2} dl(2l-L)\times [\Delta T (l,t) - \Delta T (l',t)]), \end{aligned}$$

where $n_c$ = 1.46 is the refractive index of the fiber core, $N = 48$ is the number of turns in the fiber coil, *A* is the area of the fiber coil with a diameter of 10 cm, $dn_c/dT$ = $10^{-5} / ^{\circ }$C is the temperature dependence of the refractive index of the fiber, $\alpha = 5 \times 10^{-7} / ^{\circ }$C is the coefficient of linear thermal expansion of fiberglass and *L* = 1037 m is the total length of the fiber coil. Here, *l* and $l'$ represent the locations of the two rotating beams at the same time and $\Delta T(l,t)$ and $\Delta T(l',t)$ represent the temperature differences between the locations *l* and $l'$ simultaneously.

The simplified version of the equations, simulated by using MATLAB^27^ and experimental works of thermal effects for the winding methods of four different fiber coils (dipole (AB), quadrupole (ABBA), octupole (ABBABAAB), and hexadecapole (ABBABAABBAABABBA)), are presented in the supplementary material. Besides, the rate errors caused by the stress on the fiber under varying temperature is examined and presented in (Supp. Fig. 6) in the supplementary material. Based on the outcomes, the effects coming from the stress is relatively low, hence ignored in the rest of the paper.

As seen in Supp. Eqs. (2) and (3), the rate error due to the Shupe effect is an absolute error that is independent of the direction of the real rotation. The phenomenon of the Shupe effect being independent of real rotation allows us to easily calculate the rate error due to this effect by simply reversing the fiber coil axis horizontally, such as changing its axis from the +x to the $-$x. By considering a constant rotation such as the Earth’s rotation applied to the system; the total phase shifts for the +x and $-$x axes can be shown as in Eqs. (2) and (3) ,respectively. When these phase shifts are added up to each other as shown in Eqn. (4), the twofold pure rate error due to the Shupe effect can be easily found.

2 $$\begin{aligned}{} & {} \phi _{total}^{+x} = \phi _{rotation} + \phi _{shupe_1} \end{aligned}$$

3 $$\begin{aligned}{} & {} \phi _{total}^{-x} = -\phi _{rotation} + \phi _{shupe_1} \end{aligned}$$

4 $$\begin{aligned}{} & {} \phi _{total}^{+x} +\phi _{total}^{-x} = 2\phi _{shupe_1} \end{aligned}$$

**Figure 2:**
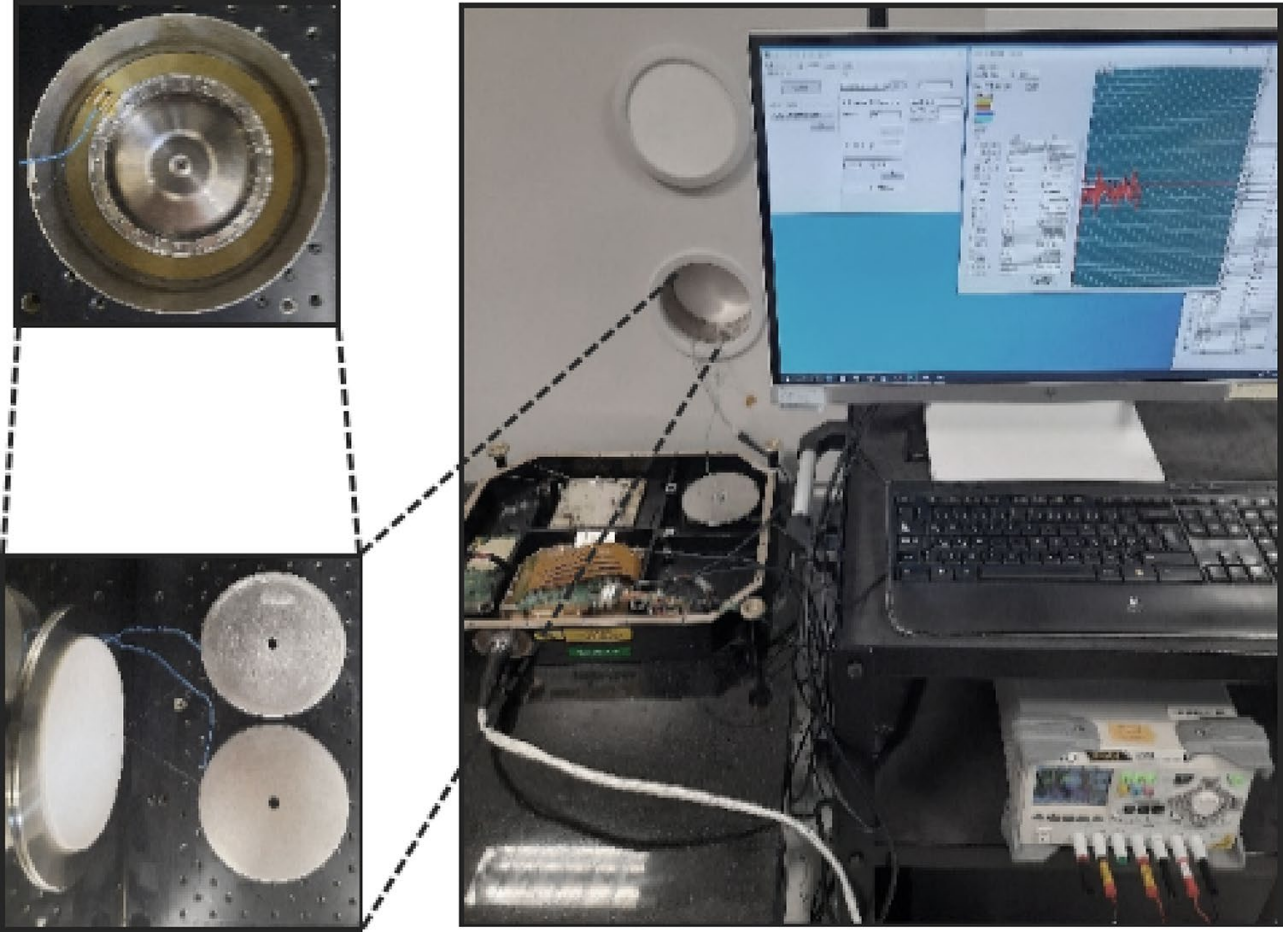
Photograph of the experimental setup.

To prove this phenomenon experimentally, we used a hexadecapolar fiber coil wound by using PM fiber with 80 $\upmu$m cladding and 168 $\upmu$m coating diameter. That fiber coil is wound on a spool with an average diameter of 85 mm consisting of 48 layers in total. Each layer had 80 turns of fiber as was also used in the model (see supplementary material). The total length of the fiber coil was approximately 1037 m and was obtained by an optical time domain reflectometer (OTDR) measurement (YOKOGAWA AQ7270). We constructed an IFOG by splicing a homemade broadband ASE light source and a three-in-one homemade multifunctional integrated optic chip (MIOC) to the fiber coil. MIOC was fabricated with annealed proton exchange (APE) method. The light source has a central wavelength of 1537 nm coupled to a 3-dB optical coupler. APE-MIOC was acting as a >40-dB polarizer, a 3-dB coupler and a phase modulator. Once the loop was completed, we implemented a photodetector to the system to convert the interfered optical signal. We packed and placed only the fiber coil with a temperature sensor in a magnetic shield to prevent nonreciprocal errors coming from magnetic field changes. First, we ran the test while the fiber coil was horizontally placed in the +x axis over different temperatures in the climatic chamber. Then, we horizontally reversed the fiber coil and repeated the same test. The rotation rate of Earth was measured as +9.6^∘^/h in Ankara, Turkey via the constructed IFOG with horizontally placed fiber coil. The rotation rate of Earth was measured as $-$9.6^∘^ /h via the constructed IFOG with horizontally reversed placed fiber coil. The rate data were read with the aid of software by using a closed-loop modulation technique and presented as a function of temperature assessed between $-$40 and +60^∘^ C operating temperature in the chamber with a 0.2^∘^C/min. rate of temperature change.

Figure 1 shows the experimental rate vs. temperature data obtained from the IFOG comprising the fiber coil placed horizontally in the +x direction (a), horizontally reversed in the $-$x direction (b) and the total phase shift in both directions (c) calculated by using Eqn. (4) from both tests respectively. To compare the rate errors due to the Shupe effect, the Earth rotation is subtracted from the real rates. Therefore, the compensated absolute rate errors that are reduced to $\pm 0.05^\circ /$h are presented with the result obtained from the IFOG comprising a perfectly trimmed hexadecapolar fiber coil (d). The comparison between these three results shows good agreement with each other.

In IFOG systems, it is not possible to simultaneously reverse the fiber coil axis horizontally. Therefore, a separate setup has been prepared to see if the presented phenomenon will still be valid when two different fiber coils are used in the setup, as shown in Fig. 2. One of the two fiber coils in this setup was horizontally reversed. The setup was prepared similarly as explained herein above. Then, the equations turn to:

5 $$\begin{aligned}{} & {} \phi _{totalC1}^{+x} = \phi _{rotation} + \phi _{shupe_{C1}} \end{aligned}$$

6 $$\begin{aligned}{} & {} \phi _{totalC2}^{-x} = -\phi _{rotation} + \phi _{shupe_{C2}} \end{aligned}$$

7 $$\begin{aligned}{} & {} R_{C1,C2} = \phi _{shupe_{C1}}/\phi _{shupe_{C2}} \end{aligned}$$

8 $$\begin{aligned}{} & {} \phi _{totalC1}^{+x} +\phi _{totalC2}^{-x} = \phi _{shupe_{C1}} + \phi _{shupe_{C2}} \end{aligned}$$

**Figure 3:**
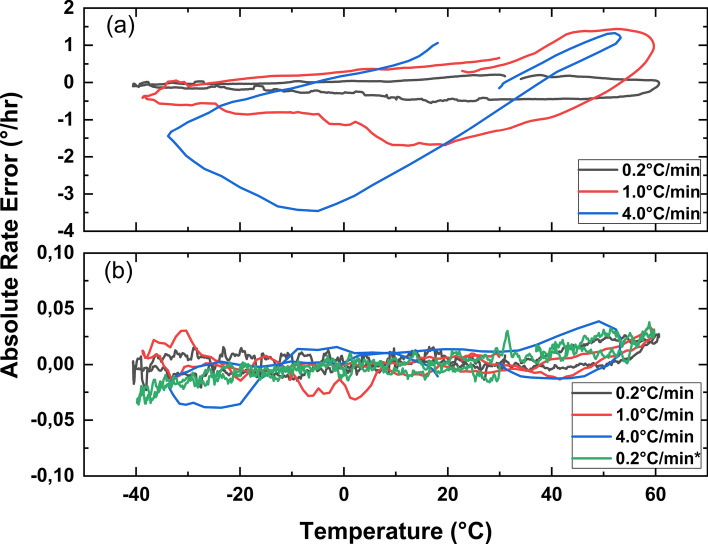
(**a**) Absolute rate errors of the IFOG comprising the hexadecapolar fiber coil obtained at a rate of 0.2^∘^ C/min., 1^∘^C/min., and 4^∘^C/min. temperature change, (**b**) compensated absolute rate errors of the hexadecapolar fiber coil, respectively, compared with the result obtained from the IFOG comprising a perfectly trimmed hexadecapolar fiber coil at a rate of a 0.2^∘^C/min. temperature change.

**Figure 4:**
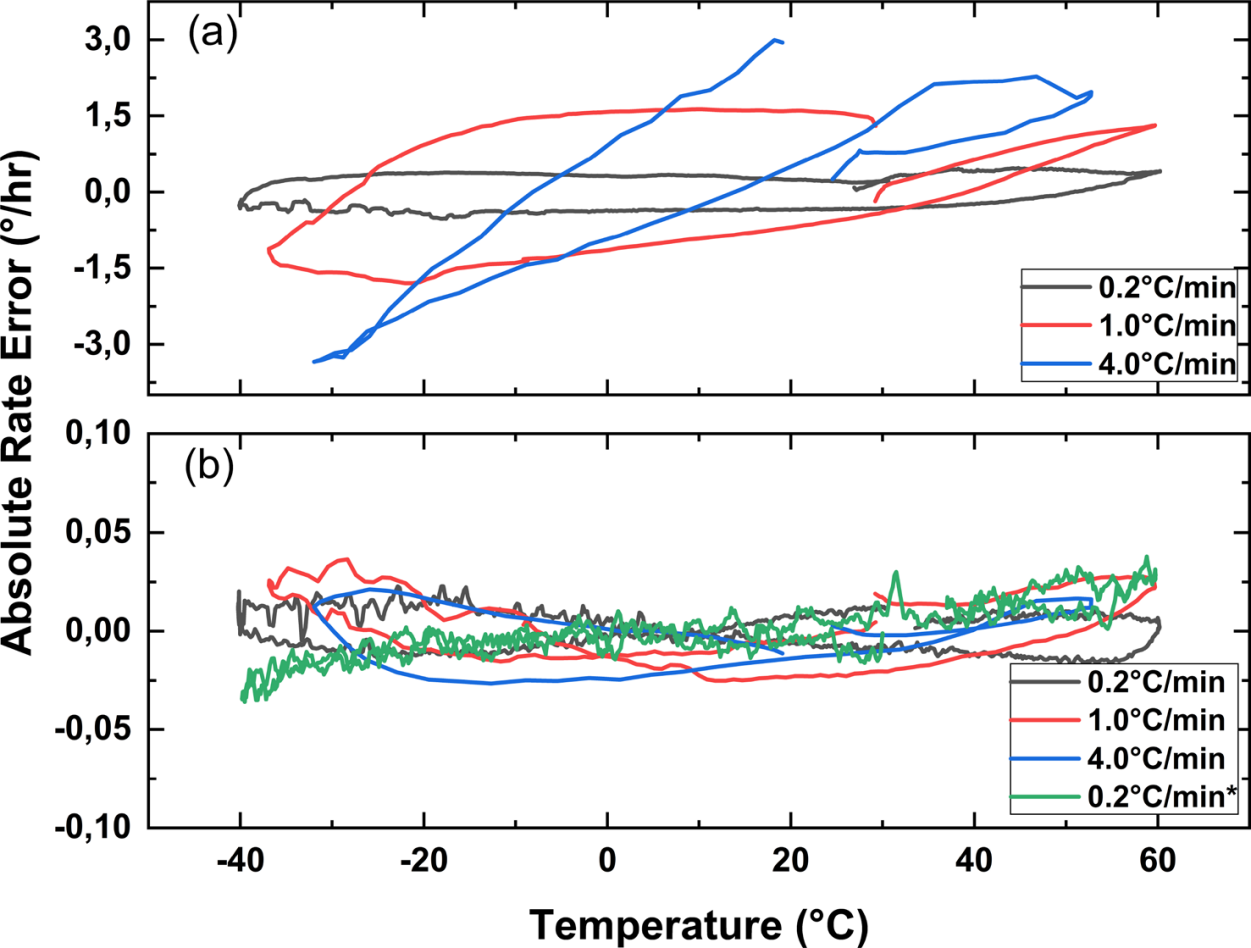
(**a**) Absolute rate errors of the IFOG comprising the quadrupolar fiber coil obtained at a rate of 0.2^∘^C/min., 1^∘^C/min., and 4^∘^C/min. temperature change, (**b**) compensated absolute rate errors of the quadrupolar fiber coil, respectively, compared with the result obtained from the IFOG comprising a perfectly trimmed hexadecapolar fiber coil at a rate of a 0.2^∘^C/min. temperature change.

If the real rotation $\phi _{rotation}$ is known, the ratio $R_{C1,C2}$ between $\phi _{shupe_1}$ and $\phi _{shupe_2}$ can be easily found by using Eqs. (5), (6), (7). Here, two different IFOGs comprising hexadecapolar fiber coils were tested at 0.2^∘^C/min., 1^∘^ C/min., and 4^∘^C/min. rates of temperature change. The ratios between these coils $R_{C1,C2}$ are found to be 3.015, 3.003, and 3.024, respectively for each rate of temperature change, under the Earth rotation. This proves that the ratio of $R_{C1,C2}$ is constant for any rate of temperature change, even if two different fiber coils are used. The uncompensated absolute rate errors are presented for one of the two different hexadecapolar fiber coils at 0.2^∘^C/min., 1^∘^C/min., and 4^∘^C/min rates of temperature in Fig. 3a. The error rate increases with respect to the rate of temperature accordingly. However, the absolute rate error can be compensated down from $\pm 2^\circ /$h (as max. rate error) to $\pm 0.05^\circ /$h as shown in Fig. 3b by using Eqs. (7), and (8). The result obtained from the IFOG comprising the perfectly trimmed hexadecapolar fiber coil at a rate of a temperature change of 0.2^∘^C/min. is also shown in Fig. 3b, and labeled as 0.2^∘^C/min.*, for comparing the results. The data obtained from the reverse axis are provided in the supplementary material (Supp. Fig. 3b).

To prove the validity of the claim about the elimination of the symmetry dependence of the fiber coils, we used one fiber coil with a hexadecapole winding pattern from the previous setup and one different fiber coil wound with a quadrupole winding pattern. We also know that fiber coils with quadrupole winding pattern is more sensitive to the Shupe effect. The ratios $R_{C1,C2}$ have also been calculated for these fiber coils. They were found to be 5.167, 5.203, and 5.189, for a 0.2^∘^C/min., 1^∘^C/min., and 4^∘^C/min. rate of temperature change, respectively. Figure 4a shows the uncompensated absolute rate error for the quadrupolar fiber coil at 0.2^∘^C/min., 1^∘^ C/min., and 4^∘^C/min. rate of temperature change. The absolute rate error can be decreased from $\pm 3^\circ /$h (as max. rate error) to $\pm 0.05^\circ /$h after the compensation as shown in Fig. 4b. The result obtained from the IFOG comprising the perfectly trimmed hexadecapolar fiber coil at a rate of a temperature change of 0.2^∘^C/min. is also shown in Fig. 4b, and labeled as 0.2^∘^C/min.* for comparing the results. The data obtained from the reverse axis are provided in the supplementary material (Supp. Fig. 4b).

**Table 1:** Comparison of different compensation methods.

Method	Temperature range (^∘^C)	Thermal transient sensitivity (^∘^/h)/(^∘^ C/min)	Fiber coil length (m)
Method 1^18^	$-$10, +50	1.130	2050
Method 2^19^	$-$40, +70	0.018	993
Method 3^20^	$-$40, +60	5.000	260
Method 4^21^	$-$40, +60	0.090	1000
Method 5^28^	$-$40, +60	0.480	1016
Proposed	$-$40, +60	0.025	1037

There are several methods used for aiming to reduce the errors caused by the Shupe effect. Table 1 shows the comparison of the proposed method with some other important ones such as modified quadrupolar fiber coil^18^, double-cylinder(D-CYL) winding^19^, crossover-free fiber optic gyros^20^, dual-polarization interferometric fiber optic gyroscope^21^, and geometric birefringence-enhanced polarization-maintaining fiber^28^ in terms of temperature range, thermal transient sensitivity and fiber coil length. According to this comparison, the proposed method shows reasonable and convincing results.

## Symmetry-independent-winding IFOG (SIW-IFOG)

**Figure 5:**
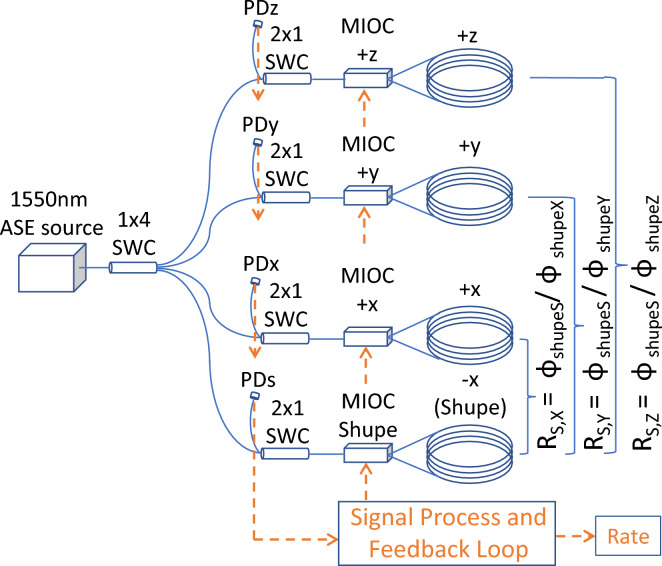
Schematics of the configuration of the symmetry-independent-winding IFOG (SIW-IFOG).

Based on the agreement that we observed between the real and the compensated results, we designed a new configuration that consists of four-axes, namely $-$x (as Shupe), +x, +y, and +z. We proposed a novel system called symmetry-independent-winding IFOG (SIW-IFOG) to compensate for the errors caused by the Shupe effect in Fig. 5. The ratios for each different fiber coil pair $R_{S,X}$, $R_{S,Y}$, and $R_{S,Z}$ can be calculated by using Eqs. (5), (6), and (7). Then, we have a constant ratio between the real rate errors coming from the Shupe effect for each different fiber coil pair, such as:

9 $$\begin{aligned} R_{S,i} = \phi _{shupeS}/\phi _{shupe(i)} \end{aligned}$$

where *i* represents the axes as X, Y, and Z according to the position of the gyroscope and the ratio $R_{S,i}$ is independent of the rate of temperature change as shown in Figs. 3 and  4.

In the system, the ratio $R_{S,X}$ between CoilS ($-$x axis) and CoilX (+x axis) will be the checkpoint to calculate how much the real rate errors are coming from the Shupe effect. By adding these two total rates, $-$x (Eqn. (11)) and +x (Eqn. (12)), the real rotations will cancel each other out and the total real rate errors will emerge, as in Eqn. (13). The known ratio $R_{S,X}$ reveals the real rate errors for the +x and $-$x axes. In addition, when the real rate error is known for CoilS, by using the ratio for remaining axes, $R_{S,Y}$, and $R_{S,Z}$, the real rate errors can also be calculated for the y and z axes easily.

10 $$\begin{aligned}{} & {} R_{S,X} = \phi _{shupeS}/\phi _{shupeX} \rightarrow known \end{aligned}$$

11 $$\begin{aligned}{} & {} \phi _{total}^{-x} = \phi _{rotation_{-x}} + \phi _{shupeS} \end{aligned}$$

12 $$\begin{aligned}{} & {} \phi _{total}^{+x} = \phi _{rotation_{+x}} + \phi _{shupeX} \end{aligned}$$

13 $$\begin{aligned}{} & {} \phi _{total}^{-x}+\phi _{total}^{+x} = \phi _{shupeS} + \phi _{shupeX} \end{aligned}$$

## Conclusion

In conclusion, we started our studies aiming to understand the thermal effects that cause phase differences other than the Sagnac effect on different fiber coils. We experimentally showed the independence of the Shupe effect on the real rotation by simply repeating the test with a single hexadecapolar fiber coil in reverse directions. We have conducted several tests at different rates of temperature changes with two IFOGs comprising different hexadecapolar fiber coils. We experimentally calculated a ratio between the real rate errors of these IFOGs. We showed that this ratio is constant for any rate of temperature change. We also reinforced our argument by repeating the test with a quadrupolar fiber coil that is known to be more sensitive to the Shupe effect. The reason behind using this fiber coil was to prove the claim about the elimination of the symmetry dependence of the fiber coil for the Shupe effect. Based on the outcomes of these studies, we proposed a novel configured complete system called SIW-IFOG that eliminates the winding symmetry dependence of the performance of the IFOGs and compensates the errors 40 times reduced for the hexadecapolar fiber coil and 60 times reduced for the quadrupolar fiber coil down to $\pm 0.05^\circ /$h that is caused by the Shupe effect. In future studies, the effectiveness of this new method will be examined for rate errors due to the Kerr effect as well as the Faraday effect under temperature change and vibration, which are independent of the direction of the real rotations.

### Supplementary Information

Supplementary Information.

## Data Availability

The datasets used and/or analysed during the current study available from the corresponding author on reasonable request.
