# Supplementary material for: A novel method to eliminate the symmetry dependence of fiber coils for shupe mitigation
Source: Sci Rep. 2024 Apr 20;14:9076. doi: 10.1038/s41598-024-59330-x (PMC11032327; doi:10.1038/s41598-024-59330-x)
Supplement: Supplementary file 1 — Supplementary Information. [file 41598_2024_59330_MOESM1_ESM.pdf]

1 Supplementary Material

We studied the thermal characteristics of fiber coils with different patterns theoretically and experimentally. We built up a numerical model and showed that the use of this winding method will help us to obtain the best thermal performance in an interferometric fiber optic gyroscope (IFOG) that consists of the respective fiber coil. We used the trimming method in addition to the usage of this special winding method for the very same purpose. We also verified the model experimentally with different rates of temperature change.

If a fiber coil experiences a temperature change over time, thermally triggered non-reciprocity arises because the two counter-propagating beams will pass through the same region of the coil at different times. The underlying reason for this effect is that when the fiber is exposed to thermal changes, the light propagation constant (β) in the fiber changes at different rates along the fiber.

Beams traveling uneven paths create undesirable nonreciprocal phase shifts along with the real phase shift coming from the rotation that is to be measured and by considering this phase shift, the angular error of thermally induced non-reciprocity for the IFOG can be calculated by [1]

$$\begin{aligned}\phi_{shupe}(t) = \quad & \frac{n_c}{4NA}(\frac{dn_c}{dT}+\alpha n_c)(\int_0^{L/2} dl(2l-L) \\ & \times[\Delta T(l,t)-\Delta T(l',t)]), \qquad (1)\end{aligned}$$

where $n_c = 1.46$ is the refractive index of the fiber core, $N = 48$ is the number of turns in the coil, A is the area of the coil with a diameter of 10 cm, $dn_c/dT = 10^{-5}/°C$ is the temperature dependence of the refractive index of the fiber, $\alpha = 5x10^{-7}/°C$ is the coefficient of linear thermal expansion of fiberglass and $L = 1037m$ is the total length of the coil. Here, l and l' represent the locations of the two rotating beams at the same time and $\Delta T(l,t)$, and $\Delta T(l',t)$ represent the temperature differences between the locations l and l' simultaneously.

After the simplification for a coil which is symmetrically wound from its midpoint, the equation becomes

$$\begin{aligned}\phi_{shupe}(t) = \quad & \gamma\sum_{i=1}^{M/2}[\sum_{j=1}^{S}[\Delta T(A_{i,j},t)-\Delta T(B_{i,j},t)] \\ & \times\int_{(i-1)L/M/S}^{iL/M/S} dl(2l-L)], \qquad (2) \\ & \gamma = \frac{n_c}{4NA}(\frac{dn_c}{dT}+\alpha n_c) \qquad (3)\end{aligned}$$

where $(A_{i,j},t)$ and $(B_{i,j},t)$ represent the clockwise and counterclockwise beam positions on the coil at the same time, $M = 48$ is the number of layers, and S is the number of dl section on each layer. This formula examines the temperature variations for each layer and each dl length corresponding to that layer. In the present paper, since the 2D

conduction thermal model is used for temperature distribution on the coil, S is taken as 80 and that also represents the number of turns for each layer of the fiber coil that is used in experiments.

Figure 1 (a) shows the geometric illustrations of the portions of repetitive sections of the fiber coils with different winding patterns along with the corresponding experimental results obtained from the IFOG comprising these fiber coils at a 0.2°C/min. rate of temperature change and Figure 1 (b-c) shows the trimming needs obtained from the calculations at 0.2°C/min. and 4°C/min. rates of temperature change. In Figure 1 (a) without trimming, 1.755, 0.356, 0.126, and 0.021°/h absolute rate errors were obtained from dipole (AB), quadrupole (ABBA), octupole (ABBABAAB) and hexadecapole (ABBABAABBAABABBA) winding patterns, respectively, at a 0.2°C/min. rate of temperature change, experimentally. Moreover, in Figure 1 (b) 2.043, 0.353, 0.100, and 0.020°/h absolute rate errors were also obtained numerically computing the results obtained from each pattern based on the previous formula showing a good agreement with the experiments. It was observed that the hexadecapole pattern outperforms the others in terms of the need for trimming as it presents approximately 100, 15, and 5 times reduced absolute rate error than dipole, quadrupole, and octupole pattern when tested under a 0.2°C/min. rate of temperature change for both simulations and experiments. As shown in Figure 1 (b-c), the need of trimming decreases as the symmetry of the winding pattern increases at both rates of temperature change. The graphs of the rate of temperature changes inside the coil for 0.2°C/min. and 4°C/min. were also given in Figure 2 with dashed lines.

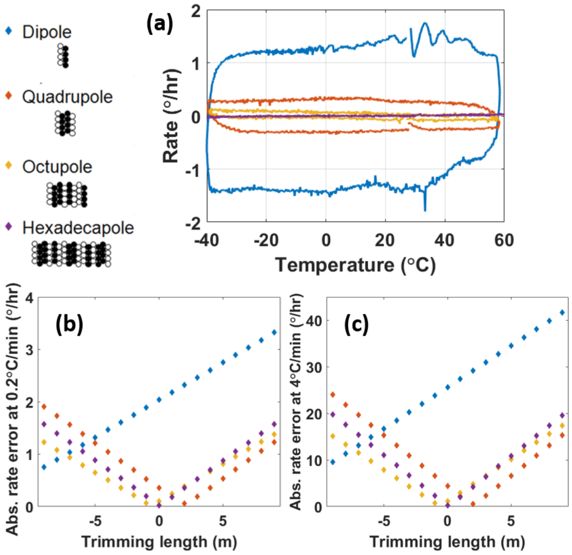

Figure 1: (a) Schematics of the winding methods of 4 different fiber coils and a comparison of the corresponding experimental rates of temperature change of 0.2°C/min. Numerical results for the absolute rate error vs. the trimming length of the fiber coils with different winding methods at a (b) 0.2°C/min. and (c) 4°C/min. rate of temperature change.

Although it was attempted to keep the winding method as symmetrical as possible, the nature of the fiber winding has a catch to it. Inevitable errors that come with the process itself such as the stress applied to the fiber during the winding, the change of the fiber length with temperature, the impurities, inhomogeneities, or the change of refractive index along the fiber [2] are always there. That is why, even if it is sought to produce a symmetrically wound fiber optic coil, the thermal performance of the fiber coil may still need to be improved at the end. Trimming is offered here as an auxiliary method to overcome this problem by basically shifting the midpoint of the fiber coil by adding or trimming a certain portion of the fiber ends [3]. In such a case, trimming shifts the midpoint of the fiber coil as $l_{eff}/2$ [4]. Then, the total angular error of thermally induced nonreciprocity including the asymmetry coming from this shift of the coil's midpoint can be calculated as

$$\begin{aligned}
\phi_{shupe}(t) = \quad & \gamma \Bigg\{ \sum_{i=1}^{M/2} \Big[\sum_{j=1}^{S} [\Delta T(A_{i,j},t) - \Delta T(B_{i,j},t)] \\
& \times \int_{(i-1)L/M/S+\frac{l_{eff}}{2}}^{iL/M/S-\frac{l_{eff}}{2}} dl(2l-L) \Big] \\
& + \sum_{i=1}^{M/2-1} \Big[\sum_{j=1}^{S} [\Delta T(A_{i,j},t) - \Delta T(B_{i,j},t)]
\end{aligned}$$

$$\times \int_{iL/M/S-\frac{l_{eff}}{2}}^{iL/M/S+\frac{l_{eff}}{2}} dl(2l-L)]\Bigg\} \tag{4}$$

The heat transfer method is used for a fixed coil geometry, material, and temperature profile to find ΔT. We use the explicit model of unsteady-state equation discretization by using a constant thermal diffusivity $\alpha = 5x10^{-9}m^2/s$ for fiberglass to calculate the temperature change in time [5].

$$\frac{\delta T}{\delta t} = \alpha(\frac{\delta^2 T}{\delta x^2} + \frac{\delta^2 T}{\delta y^2}) \tag{5}$$

After the discretization of the equation, it becomes

$$\frac{T_M^{n+1} - T_M^n}{\Delta t} = \alpha(\frac{T_L - 2T_M + T_R}{\delta x^2} + \frac{T_T - 2T_M + T_B}{\delta y^2}) \tag{6}$$

$$\left\{T_M^{n+1} = T_M^n + k_1(T_L - 2T_M + T_R)^n + k_2(T_T - 2T_M + T_B)^n\right\} \tag{7}$$

where $k_1 = \alpha\Delta t/\Delta x^2$ and $k_2 = \alpha\Delta t/\Delta y^2$, T_L, T_T, T_M, T_B, T_R represent left, top, middle, bottom, and right boundary respectively if the conductivity $k_{1,2}$ were to be constant over the region.

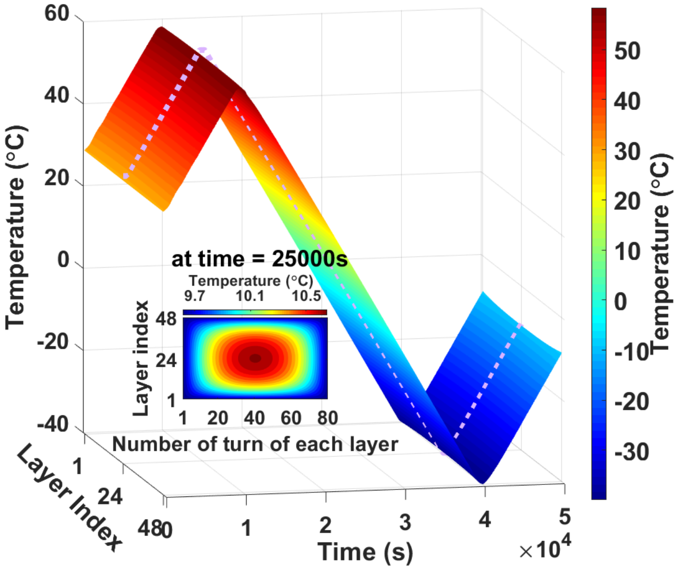

(a) Rate of temperature change 0.2°C/min.

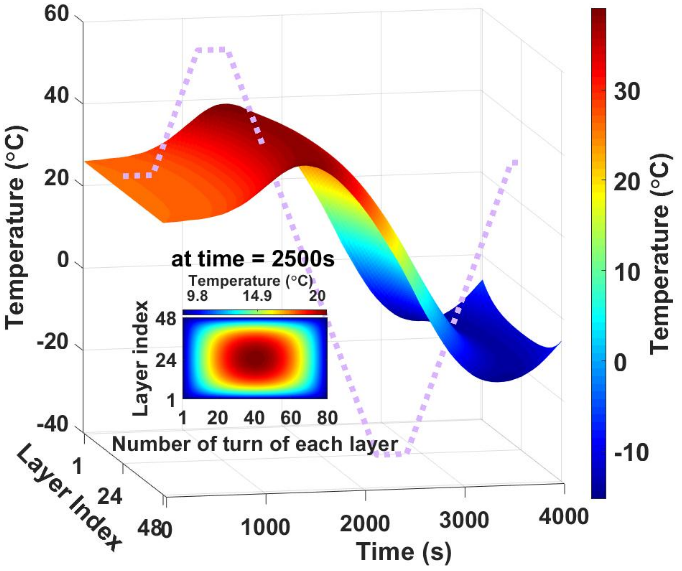

(b) Rate of temperature change 4°C/min.

Figure 2: Temperature distribution curves on each layer of the fiber coil with respect to time at different rates of temperature change and heat transfers of the layers with respect to the number of turns of each layer at certain times.

Figure 2 shows the temperature distributions on each layer of the fiber coil with respect to the time and layer index at different rates of temperature change. Dashed lines indicate the set temperature of the climatic chamber where the fiber coil is tested. The cross-sections of the layers and turns of the fiber coil at certain times are also given as a function of temperature. While the rate of temperature change is low, e.g., 0.2°C/min., the set temperature and the temperature felt by the coil almost match and the temperature difference between the coldest and the hottest point of the fiber coil becomes 1.1°C at t=25000s. However, when we increase the rate of the temperature change up to 4°C/min., the set temperature is no longer felt by the fiber coil properly and the temperature difference between the coldest and the hottest point of the fiber coil becomes 10.5°C at t=2500s. The temperature variation itself among the layers also varies upon different rates of temperature change.

We observed that the Shupe effect is independent of the direction of the real rotation as shown in Figure 1 in the main article. We kept running the tests by using pairs of hexadecapolar and quadrupolar fiber coils at a different rate of temperature changes in order to further investigate the thermal effects on different fiber coils as shown in Figure 3 and Figure 4. We used the experimental setup of which details are described in the main article.

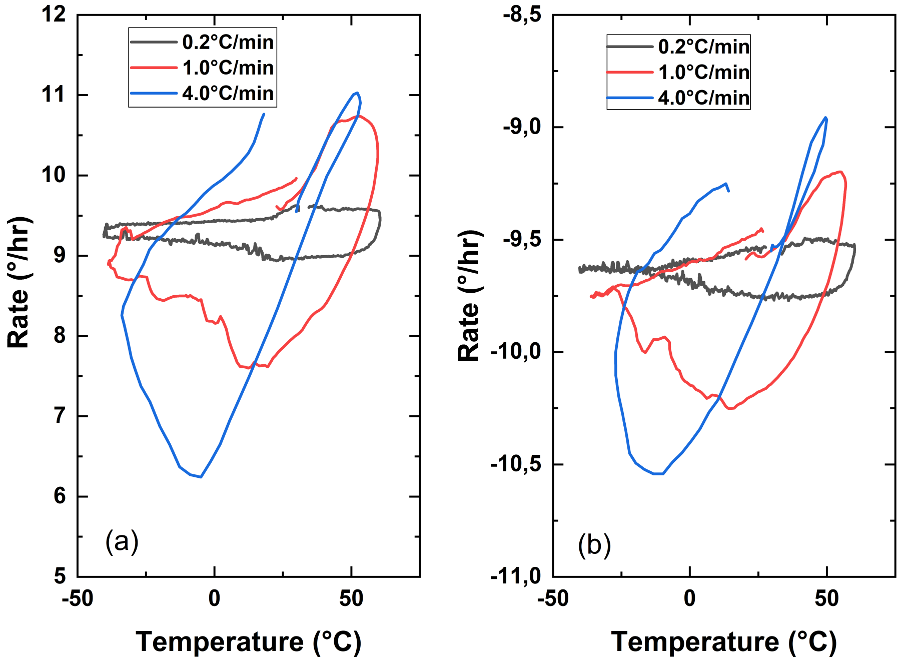

Figure 3: Rate vs. temperature graphs of the IFOGs comprising (a) the horizontally placed hexadecapolar fiber coil, (b) horizontally reversed placed hexadecapolar fiber coil at a rate of 0.2°C/min., 1°C/min., and 4°C/min. temperature change

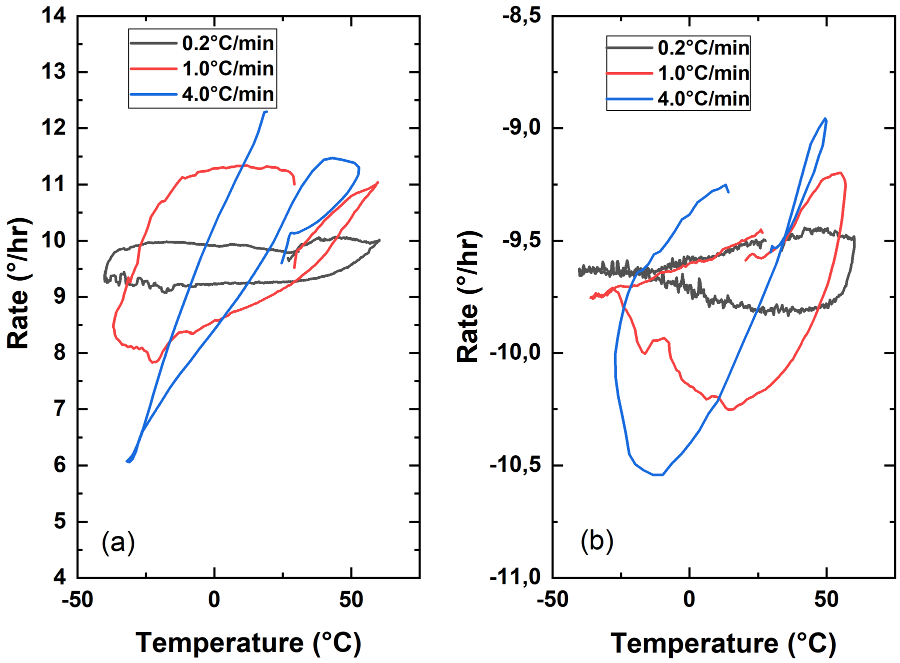

Figure 4: Rate vs. temperature graphs of the IFOGs comprising (a) the horizontally placed quadrupolar fiber coil, (b) horizontally reversed placed hexadecapolar fiber coil at a rate of 0.2°C/min., 1°C/min., and 4°C/min. temperature change

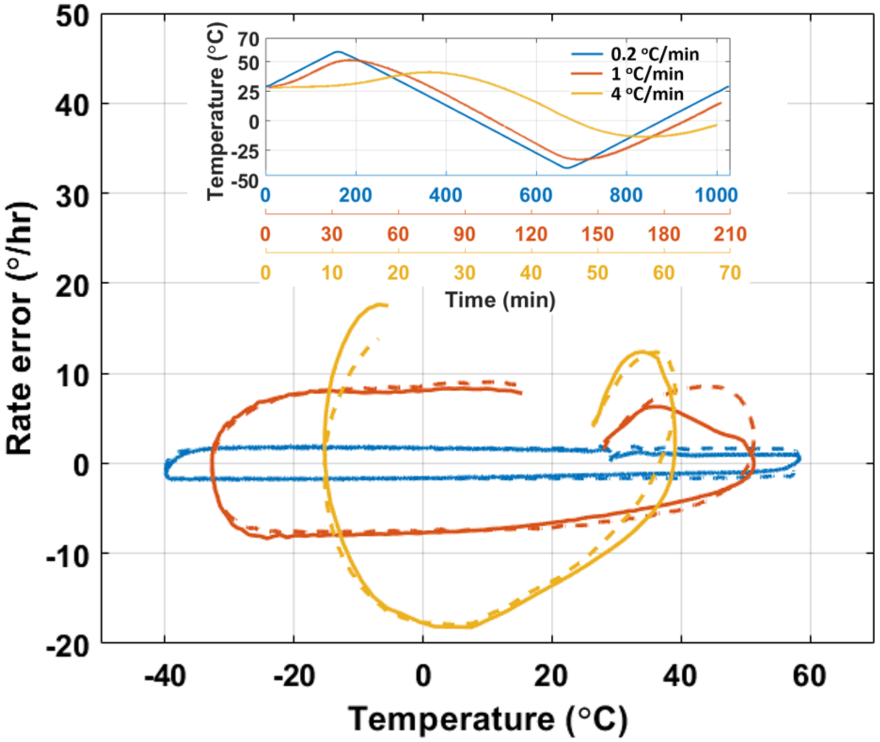

Figure 5: Comparison of the rate data (around zero) of the IFOG comprising the fiber coil with the hexadecapole pattern with different rates of temperature change (inset-sensor reading on coil) obtained from experiments (solid lines) and simulations (dashed lines) at a 10 m trimming length.

Figure 5 shows the experimental and simulated absolute rate errors at three different rates of temperature change of 0.2, 1, and 4°C/min. at 10 m trimming length. 1.704°/h absolute rate error was measured at a rate of 0.2°C/min. temperature change with both experiments and simulations. 8.239°/h and 9.007°/h at a rate of 1°C/min., 17.99°/h and 18.15°/h at a rate of 4°C/min. temperature change with experiments and simulations, respectively.

The simulation used in Figure 5 only shows the error due to the Shupe effect which is related to temperature dependence of fiber refractive index and thermal expansion of the fiber. In addition, the rate error due to stress in the core and cladding resulting from temperature changes was examined in Figure 6. However, as can be seen in Figure 6, this effect turned out to be much lower than the Shupe effect. In Eqn. 8, the rate error due to stress varies depending on ΔT rather than the change of ΔT in time. As proof of this, as the rate of temperature change increases, the stress-related rate error also decreases. The reason behind this is the fact that the temperature of the fiber on the coil changes before reaching the minimum and maximum values in high-rate of temperature changes. As a result of rate errors caused by stress remaining very low in

the simulations, the simulations were continued with the error rates caused only by the Shupe effect.

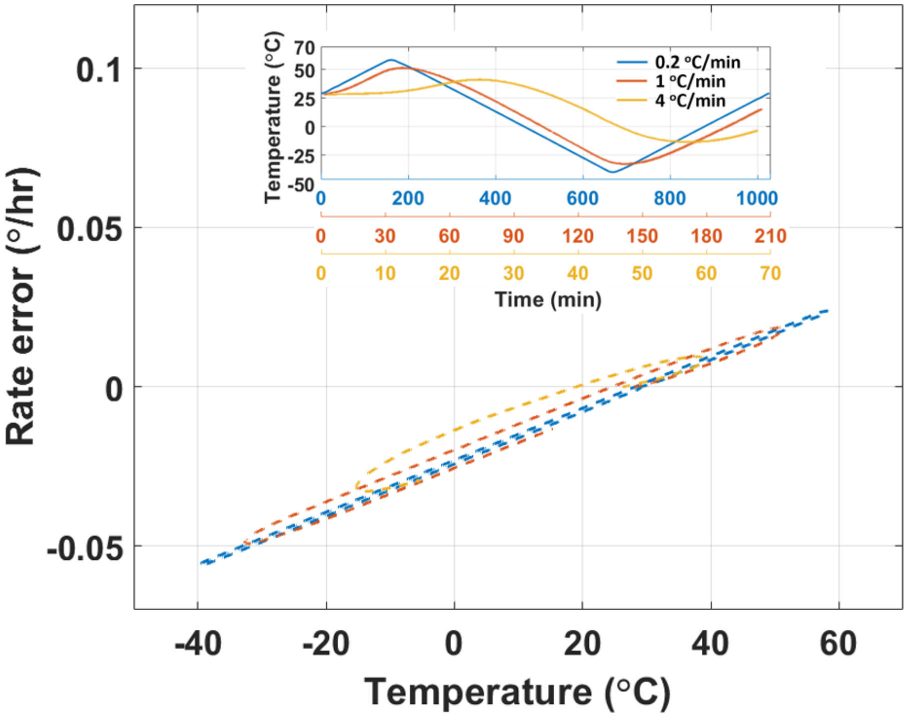

Figure 6: The simulated rate data errors due to stress (around zero) of the IFOG comprising the fiber coil with the hexadecapole pattern with different rates of temperature change (inset-sensor reading on coil)

$$\begin{aligned}\phi_{stress}(t) = \quad & \frac{c}{DL}\left(\int_0^L \left[\frac{2n\mu}{E_{core}} + \frac{n^3}{2E_{core}}(P_{11} - \mu P_{11} + P_{12} - 3\mu P_{12})\right]\right. \\ & \times [E_{coating} \cdot \alpha_c \cdot \Delta T(t)] dl, \end{aligned} \tag{8}$$

where c is the speed of light, $D = 10cm$ is the diameter of the coil, $L = 1037m$ is the length of the fiber coil, $n = 1.45$ is the refractive index of the fiber. The terms in the first bracket are related to rate error with thermal stress [6] and μ, E_{core}, P_{11} and P_{12} are Poisson's ratio, Young modulus of the fiber core and photoelastic coefficients respectively. The second bracket is related to thermal stress induced by fiber coating expansion and $E_{coating}$, α_c are Young's modulus and thermal expansion coefficient of the fiber coating respectively. $\Delta T(t)$ is the variation of the temperature. The parameters for the calculation are taken from the paper [6].

The agreement of the simulated and experimental results at different rates of temperature changes supports the reliability of the thermal model that we used. Based

on this, we investigated the effect of the trimming length of the fiber coil on the absolute rate error. Figure 7 shows the relation between the absolute rate errors with respect to the trimming length of the fiber coil at different rates of temperature change. 0.023°/h absolute rate error was obtained with simulations indicated with dashed lines and 0.024°/h absolute rate error was obtained experimentally indicated with dots at the rate of temperature change of 0.2°C/min. Likewise, 0.023°/h absolute rate error was obtained with simulations and 0.035°/h absolute rate error was obtained experimentally at a rate of temperature change of 4°C/min. showing a good agreement with each other. In order to obtain these results, we still need to find and adjust the perfect trimming amount on the fiber coil at the order of 10 cm length. There, the trimming length is directly proportional to the absolute rate error. Therefore, increasing the trimming length in such coils worsens the thermal performance of the IFOG comprising the corresponding fiber coil as it increases the absolute rate error. In addition to this, as shown in Figure 7, there is a clear slope difference between shorter and longer trimming lengths at about 20 m and that is because the trimming length is longer than about 20 m that is longer than one layer of the coil that is used in the setup, causing a change in the symmetry of the hexadecapole pattern.

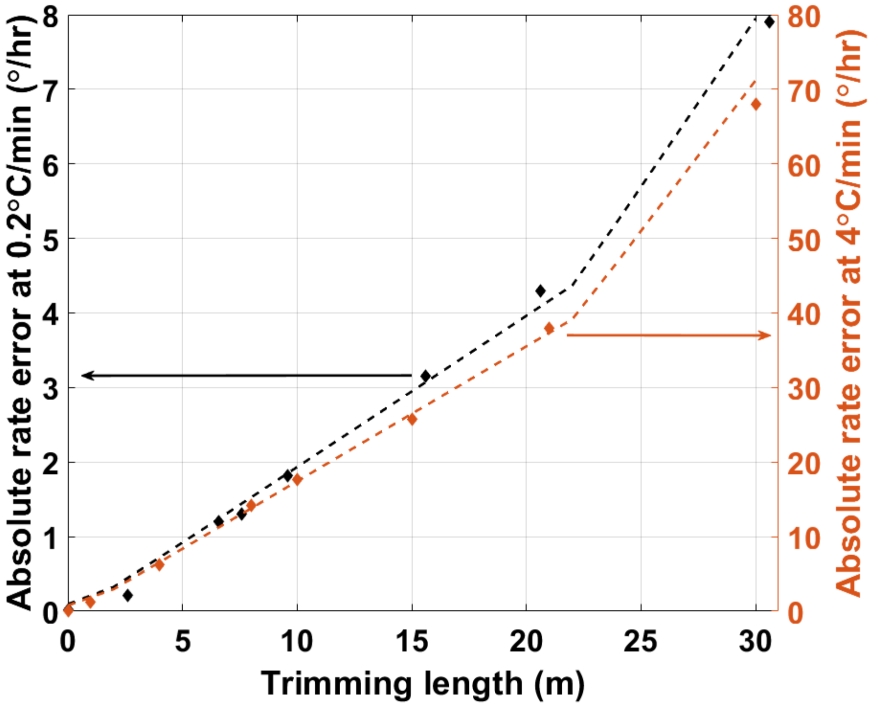

Figure 7: Comparison of the experimental (dots) and simulated (dashes) absolute rate errors at the rates of temperature change of 0.2°C/min. and 4°C/min. with respect to the trimming length.

References

[1] David M Shupe. “Thermally induced nonreciprocity in the fiber-optic interferometer”. In: *Applied optics* 19.5 (1980), pp. 654–655.

[2] Zhuo Zhang and Fei Yu. “Quantitative analysis for the effect of the thermal physical property parameter of adhesive on the thermal performance of the quadrupolar fiber coil”. In: *Optics express* 25.24 (2017), pp. 30513–30525.

[3] Randy P Goettsche and Ralph A Bergh. *Trimming of fiber optic winding and method of achieving same*. US Patent 5,528,715. June 1996.

[4] Zhihong Li et al. “A novel method for determining and improving the quality of a quadrupolar fiber gyro coil under temperature variations”. In: *Optics Express* 21.2 (2013), pp. 2521–2530.

[5] Mary Remley Albert and Gary E Phetteplace. “Computer models for two-dimensional steady-state heat conduction”. In: *Computer models for two-dimensional steady-state heat conduction* (1983).

[6] Aixi Zhang. “Student Member, IEEE, Lining Zhang, Member, IEEE, Zhikai Tang, Student Member, IEEE, Xiaoxu Cheng, Yan Wang, Kevin J. Chen, Fellow, IEEE, and Mansun Chan, Fellow, IEEE, Analytical Modeling of Capacitances for GaN HEMTs, Including Parasitic Components”. In: *IEEE Transactions on Electron Devices* 61.3 (2014).
